# Maximizing the Recycling of Iron Ore Pellets Fines Using Innovative Organic Binders

**DOI:** 10.3390/ma16103888

**Published:** 2023-05-22

**Authors:** Karthik Manu, Elsayed Mousa, Hesham Ahmed, Mohamed Elsadek, Weihong Yang

**Affiliations:** 1 Department of Material Science and Engineering, KTH Royal Institute of Technology, 100 44 Stockholm, Sweden; 2 SWERIM AB, Aronstorpsvägen 1, 974 37 Luleå, Sweden; 3 Central Metallurgical Research and Development Institute (CMRDI), Cairo 12422, Egypt; 4 MiMeR—Minerals and Metallurgical Engineering, Luleå University of Technology, 971 87 Luleå, Sweden

**Keywords:** agglomeration, pellet fines, organic binders, briquettes, reduction, green steel

## Abstract

This research work focuses on the practicality of using organic binders for the briquetting of pellet fines. The developed briquettes were evaluated in terms of mechanical strength and reduction behavior with hydrogen. A hydraulic compression testing machine and thermogravimetric analysis were incorporated into this work to investigate the mechanical strength and reduction behavior of the produced briquettes. Six organic binders, namely Kempel, lignin, starch, lignosulfonate, Alcotac CB6, and Alcotac FE14, in addition to sodium silicate, were tested for the briquetting of pellet fines. The highest mechanical strength was achieved using sodium silicate, Kempel, CB6, and lignosulfonate. The best combination of binder to attain the required mechanical strength even after 100% reduction was found to be a combination of 1.5 wt.% of organic binder (either CB6 or Kempel) with 0.5 wt.% of inorganic binder (sodium silicate). Upscaling using an extruder gave propitious results in the reduction behavior, as the produced briquettes were highly porous and attained pre-requisite mechanical strength.

## 1. Introduction

The iron and steel industry is considered to be the backbone of industrialization. Traditionally, fine ores are treated either by the process of sintering and/or by pelletizing to prepare an agglomerate with suitable metallurgical characteristics for ironmaking processes. The study put forward by [1] compared sinter to pellets and clearly stated various environmental and technical benefits of using pellets instead of sinters. To increase the gas permeability in the shaft and the furnace efficiency during the direct reduction process, pellets are the preferred feed when in comparison with lump ore [2].

Inevitable generation of pellet fines mainly occurs during the transportation, handling, or screening of pellets before charging them to the shaft furnace. Previous studies [3,4] suggest that around 24 million tons of pellet fines are generated all around the world by the year 2020. Parallelly, utilizing these generated pellet fines is of utmost importance to increase resource efficiency, thus ensuring the conservation of natural resources. Generally, the generated fines are less than 8 mm in size and possess more than 65 wt.% of iron content [5]. Aside from saving the generated pellet fines, there is a need to agglomerate these fines into the form of briquettes or pellets before charging them to the furnace. One of the main reasons for agglomeration is to maintain the size and, hence, facilitate the reduction of gas flow through the charge uniformly at a high rate. Fines charge will give rise to a non-permeable bed and is susceptible to getting carried away during high flow rates. Therefore, to increase the permeability and to limit the material blowing out of the furnace as dust, various agglomeration methods have been adopted, of which the most commonly used ones are sintering, pelletizing, and briquetting [6]. Briquetting is the process of compaction of fines into chunks of regular shape with the use of a vibro-press, piston press, extruder, or roller press [7]. By preparing the fine ores by the method of pelletization or briquetting, gaseous emissions are lowered, in comparison with the sintering route. Pelletization process is preferred for the agglomeration of very fine particles, of micron ranges [8]. Hence, briquetting will be the best type of agglomeration for coarser particles [5,9]. Furthermore, variables such as binder type, binder dose, compaction pressure, mixing, surface characteristics, and size distribution of raw material influence the final quality of the processed briquette [10].

Nowadays, the iron ore pellet fines are used as a part of the sinter mixture during the sintering process to produce sinter for the blast furnace. In Nordic countries (e.g., Sweden and Finland) where sintering is not available anymore, the pellet fines are recycled into a blast furnace, thereby mixing it with other steel mill residues and briquetting in form of hexagonal shape briquette using a vibro-press. However, the main problem arises as these two uses are becoming obsolete since they are not suitable for the hydrogen-based direct reduction process.

Around 500 kg of waste (solid by-products such as slags) are generated for each ton production of crude steel [11]. Replacement of traditionally used inorganic binders with an organic binder for briquetting of pellet fines will reduce the amount of gangue constituents (such as alumina, silica, and calcium) in the final product, which should be limited to an amount lesser than 2 wt.% [12]. Furthermore, the incorporation of organic binders gives rise to better reducibility even though the strength is compromised. The usage of organic binders contributes to shorter diffusion paths, thereby introducing a greater number of reduction sites in the material simultaneously [13], whereas the use of organic binder alone is not preferred because of their property of high decomposition rate when subjected to high temperature [14]. Hence, the most plausible way to obtain briquettes that can sustain material attenuation due to attrition is to design the mix with a combination of both organic and inorganic binders.

The potential of agglomerating pellet fines with organic and inorganic binders needs to be analyzed as the first part. Hence, the current work mainly concentrates on the potential of recycling disregarded pellet fines for iron production by agglomerating with an organic binder, without compromising the mechanical and reduction properties of the agglomerates. The effect of reduction with hydrogen is also explored in this study, so as to check whether the produced briquettes fulfill the pre-requisite strength before and after reduction. Furthermore, a way to upscale the briquette production using an extruder is tested and the produced briquettes were subjected to strength and reduction tests.

## 2. Materials and Methods

### 2.1. Materials and Sample Preparation

Pellet fines were supplied in powdered form, which required further analysis. The supplied pellet fines were characterized according to their moisture content and the chemical composition of various elements present. By reviewing previous research works and recommendations given by binder developers, six organic binders (see Table 1) along with one inorganic binder (sodium silicate trihydrate, Na_2_SiO_3_·3H_2_O), were selected for further analysis and testing. Primarily, screening of binders should be done to select the most promising binders among the selected ones.

In all experimental research work involving powdered materials, sample preparation is of utmost importance to obtain a consistent mixture. In this work, the supplied pellet fines needed to be prepared to select a representative sample for further analysis. This was achieved by mixing the entire amount of supplied material with the help of an Eirich intensive mixer, which is a compact equipment used to acquire a homogeneous mixture whenever the material is supplied in mass quantity and is difficult to mix the sample manually. Chemical and physical characterization was done for iron ore pellets fines and developed briquettes, as briefly described in the following sub-sections.

#### 2.1.1. Chemical and Phase Analysis

Chemical analysis of the supplied pellet fines was conducted using X-ray Fluorescence (XRF) technique to understand the chemical composition of various elements present in the supplied sample. To check for the emissions from the briquettes, the carbon and sulfur content in the pellet fines was examined using LECO CS230 analysis. Phase analysis was performed using X-ray diffractometer (XRD). A Panalytical Empyrean XRD (Malvern Panalytical B.V., Almelo, The Netherlands) in θ-θ geometry with Cu Kα radiation (λ = 0.154184 nm), a beam current of 40 mA and beam voltage of 45 mV was used to determine the variation in phase composition with respect to reduction extent.

#### 2.1.2. Particle Size Distribution

Particle size largely influences the surface area, compaction, and mechanical properties of the final material. The extent of finer particles influences the degree of densification for the processed briquettes. In the experiments of [11], it was also proved that the stability and homogeneity of the produced briquettes will be adversely affected while incorporating the use of bigger size fractions (>5 mm) of raw materials during the process of briquetting. Hence, to attain an overview of the size ranges of the sample, a mechanical sieve shaker (Retsch AS200 basic) was incorporated in this work to determine the particle size distribution. To determine the size ranges, sieves of different sizes (0.063, 0.25, 0.5, 1, 2, 2.8, 4, 5.6, 7.1, 10, and 11.2 mm) were stacked upon in increasing order, from bottom to top, and a proper shaking time (5–10 min) was given so as to ensure proper settling down of the powdered sample material.

#### 2.1.3. Moisture Content Analysis

The processability and strength of the developed briquettes largely depend upon the moisture content in them and, hence, it is required to determine the initial moisture content in all of the samples and binders, so as to add up to the total moisture content in the produced briquette. According to the experiment conducted by [15], the amount of initial moisture present in the sample affects the shrinkage property, which in turn affects the strength of the final product [4,9]. All of the individual moisture content within each material was determined by precisely weighing and loading it to a Mettler Toledo moisture analyzer (Mettler-Toledo AG Laboratory & Weighing Technologies, Greifensee, Switzerland) with a halogen heating unit. 

### 2.2. Briquetting and Testing

In this study, cylindrical shape briquettes were produced using a hydraulic piston press (Herzog, HERZOG Maschinenfabrik GmbH & Co. KG, Osnabrück, Germany). An equal weight (~20 g) was loaded into the mold (diameter = 20 mm) for each pass. Briquetting was done at different compaction levels (50–200 kN) to investigate the effect of pressure on the briquette strength. Thereafter, each briquette was tested using a hydraulic compression testing machine (ENERPAC Applied Power GmbH, Düsseldorf, Germany) for obtaining their Cold Compressive Strength (CCS) and Splitting Tensile Strength (STS). To measure the compressive and splitting strength of the briquettes, they were placed on a designated metal base and subjected to compression using a mobile probe with a velocity of around 20 mm/min. As the load on the briquette increased, the compressive tester machine automatically recorded the corresponding compression force values in Newtons (N). The briquettes were compressed in the longitude position so as to test for the CCS, while each briquette was compressed in the radial direction during STS measurement. The tests were performed in accordance with ISO 4700:2007 standard [16]. In order to obtain credible strength values, three briquettes were tested for each CCS and STS measurement, which were then averaged. After every compaction of different recipes, disintegrated briquettes were collected and tested for moisture content for further analysis.

### 2.3. Design of Experiments

Design of Experiments (DoE) is an approach used when conducting an experiment in which several parameters need to be considered and optimized. This experimental study, in turn, deals with many parameters and it is necessary to determine their effects on strength. The program MODDE 13, by Sartorius Stedim Data Analytics AB (Sartorius Lab Instruments GmbH & Co. KG, Goettingen, Germany) was used. In order to set up the DOE, we started by identifying the factors, responses, and limits of the process. The influencing parameters were binder percentage, compaction pressure, and moisture content. Thereafter, parameters of interest were entered, and their respective requirements were entered so as to obtain a sweet spot contour plot. The sweet spot is the area in the plot where all the required criteria are met. DoE analysis, thus, gives the optimum condition for producing a briquette with the maximum compressive and splitting strength. One of the main abbreviations is the use of S.S instead of sodium silicate. Other abbreviations that are being used throughout the workare shown in Table 2. 

### 2.4. Reduction of Developed Briquettes

Reduction progression was monitored using Thermogravimetric analysis (TGA), which was performed using a Netzsch STA 409 instrument (Erich NETZSCH GmbH & Co. Holding KG, Selb, Germany). Briquette stability and the amount of volatile component were determined by monitoring the weight change of the samples when heated at a constant rate of 20 °C/min until the temperature reached 950 °C. The experiment was continued until the required reduction extent was achieved (25%, 50%, 75%, or 100%). Hydrogen was made to be the reducing gas and a flow rate of 100 mL/min was selected for the analysis. Interrupted reduction tests were also performed to check the variation in mass loss percentage and strength variation at different reduction extents. Thereafter, strength tests for the samples after the reduction were crosschecked with the required magnitude of compressive strength, that is, 15–20 kg/cm^2^, as suggested by previous research work [12].

### 2.5. Upscaling Trials

Upscaling is of interest when it comes to industrial trials. Upscaling of work is, hence, required so as to confirm that the same promising results can also be obtained during large-scale trials. In this work, large-scale production of briquettes was facilitated with the use of an extruder (Mod. DEX-80, Tallers Felipe Verdés, Barcelona, Spain) using 20 mm steel die for extruded pellets. By making use of an extruder, briquettes with lower compaction pressure can be made at a higher production rate. The recipe made of the binder that possesses the best green compressive strength was selected for the extrusion since the extruded briquettes are highly susceptible to breaking during handling. Recipes were then tested for compression and splitting tensile strength in order to obtain a comparison over the labor-intensive hand press briquetting. Drop test and the reducing behavior for the extruded briquettes were also analyzed at a later stage. 

## 3. Results and Discussion

### 3.1. Sample Characterization

#### 3.1.1. Chemical Analysis

Chemical analysis of the supplied pellet fines was determined using XRF technique and all of the individual element percentages were analyzed. It was confirmed that the major constituent of pellet fines was hematite (Fe_2_O_3_) being 95.11 wt.% along with minute fractions of CaO, SiO_2_, MnO, MgO, Al_2_O_3_ (of the range ~1 wt.%).

#### 3.1.2. Particle Size Distribution

**Figure 1:**
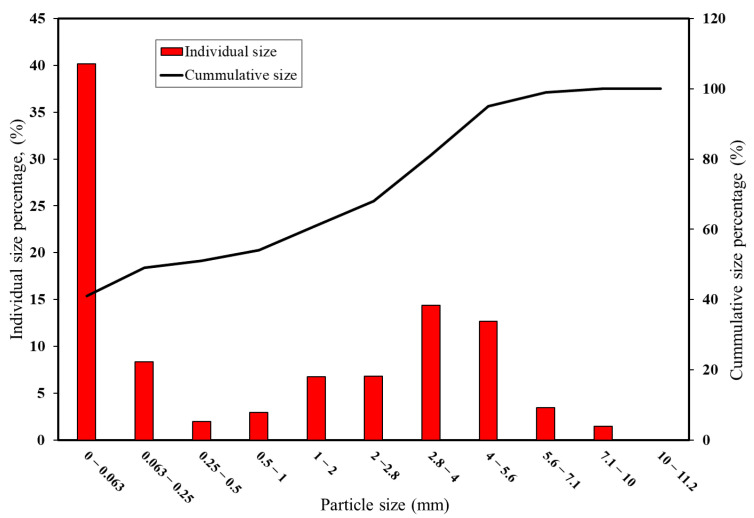
Particle size distribution of the supplied pellet fines.

The usage of a mechanical sieve shaker for the supplied pellet fines gave the particle size distribution as shown in Figure 1. It can be inferred that the pellet fines are mainly composed of very fine material below 0.063 mm in size. Since it involves a larger fraction of material between 1.0 and 5.6 mm in size range, briquetting is the dominating agglomeration technique that can be used in this case, when in comparison with the pelletizing process.

#### 3.1.3. Moisture Content

Moisture analysis of pellet fines confirmed that they are supplied in dry form, with a very limited amount of moisture content (0.03%), whereas binders had a relatively high moisture content (5–10%).

### 3.2. Screening of Binders

To derive a correlation between the selected binder performances, all of the seven binders were weighed 1 wt.% of the total weight (20 g) for the production of briquettes by manual hand pressing. Pre-designed recipes for the purpose of screening binders are shown in Table 3. The effect of all seven selected binders on the strength of the developed briquettes was determined by comparing the CCS and STS of each individual briquette. Eight recipes were produced using pellet fines as the matrix. R0 is made to be the reference recipe, a recipe comprising only pellet fines, i.e., no binder. CCS and STS tests were performed directly after compression, after 24 h, 96 h, 168 h, and after drying the sample in the oven at 105 °C for 2 h. To optimize the amount of water that needs to be added to the mixture for obtaining high mechanical properties, the moisture content in the briquette was also measured.

**Figure 2:**
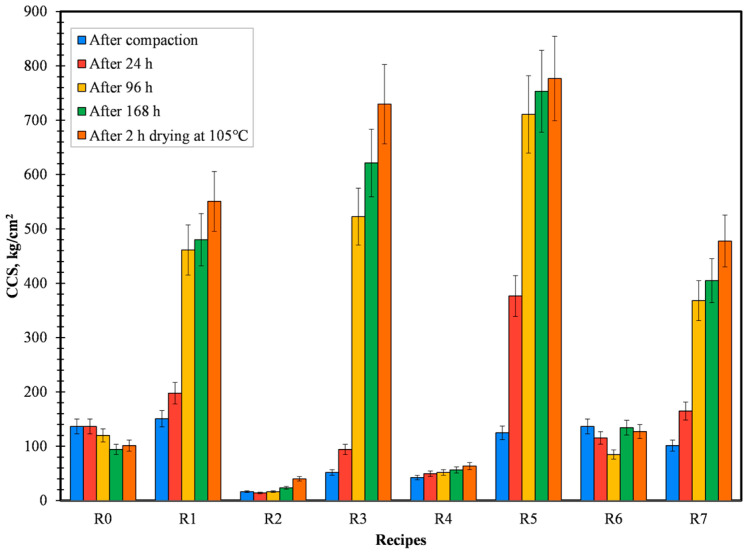
Variation in CCS for all the produced recipes.

**Figure 3:**
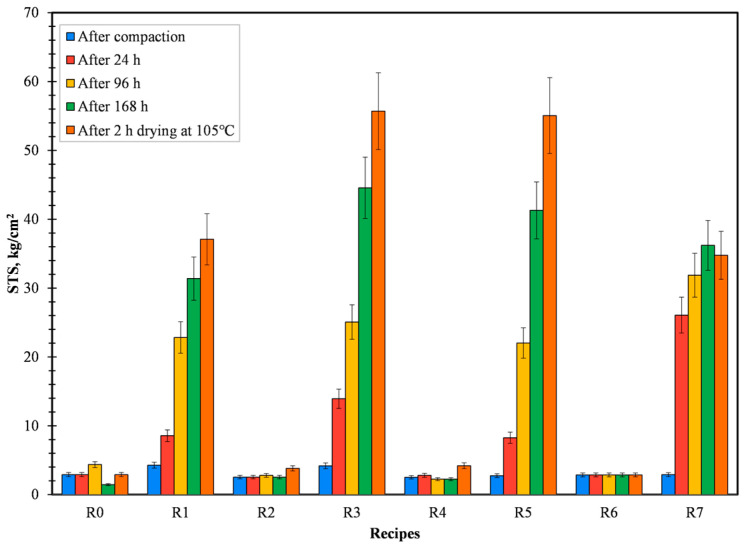
Variation in STS for all the produced recipes.

From Figure 2 and Figure 3, it can be ascertained that the recipes R1 (CB6), R3 (Kempel), R5 (sodium silicate), and R7 (lignosulfonate) are able to give high dry compressive and splitting strength when in comparison with other binders. Good densification of briquettes (briquettes with significantly less disintegration of material after production) was achieved because of the high compaction pressure of 200 KN and due to the presence of finer particles in the prime material, i.e., pellet fines. From Figure 2 and Figure 3, it can also be confirmed that the reference recipe depicted a loss of strength when left to dry either in the air or oven. This trend can be attributed to the lack of binders to improve the cohesion force between particles when water is introduced. In the case of R1, R3, R5, and R7, all of the binders efficiently reacted with water and developed strong cohesion force in between particles, consequently increasing the CCS and STS of the briquettes. In parallel to that trend, briquettes dried in the oven at 105 °C for 2 h possessed higher strength when in comparison with air-dried briquettes, i.e., Drying Compressive Strength (DCS) or Drying Splitting Strength (DSS) were found to be dominant with respect to Air Compressive Strength (ACS) or Air Splitting Strength (ASS).

Green compressive and splitting strength is of utmost importance in order to sustain the minor disintegration of briquettes directly after the production process [17]. Taking green and drying compressive strength into account, recipes R1, R3, R5, and R7 proved to be the best. For the ease of experiments, only CB6, Kempel, and sodium silicate were selected for further evaluation. In spite of that, using lignosulfonate as an organic binder is an area to study further as it is 100% renewable [18].

### 3.3. Design of Experiments Results

The selected three binders (CB6, Kempel, and sodium silicate), which were found to be the best out of eight recipes, were fed into the DOE software (MODDE version 13), and the total number of necessary experimental runs was determined. Preliminary experiments with 0–10 wt.% of water proved that 5 wt.% of water is the optimum condition for briquetting. Whenever the water content was above 5 wt.%, excess water was found to squeeze out from the mold during briquetting. Hence, for the purpose of DOE, the moisture content in the mixture is made to vary from 0 to 5 wt.%, compaction pressure from 50 to 200 kN, and binder content from 0 to 2 wt.%. Table 4 gives the composition of all the recipes that were produced to determine the optimum condition for maximum compressive and splitting strength.

**Figure 4:**
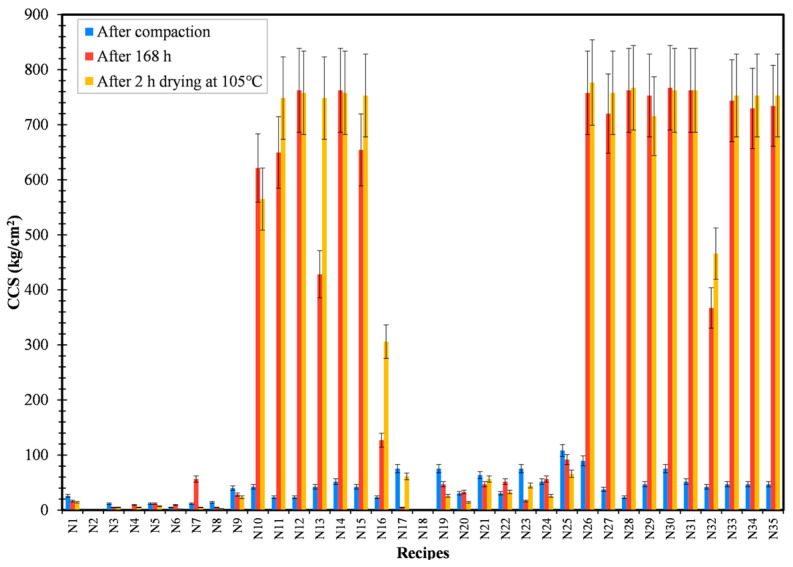
Variation of CCS for developed recipes.

**Figure 5:**
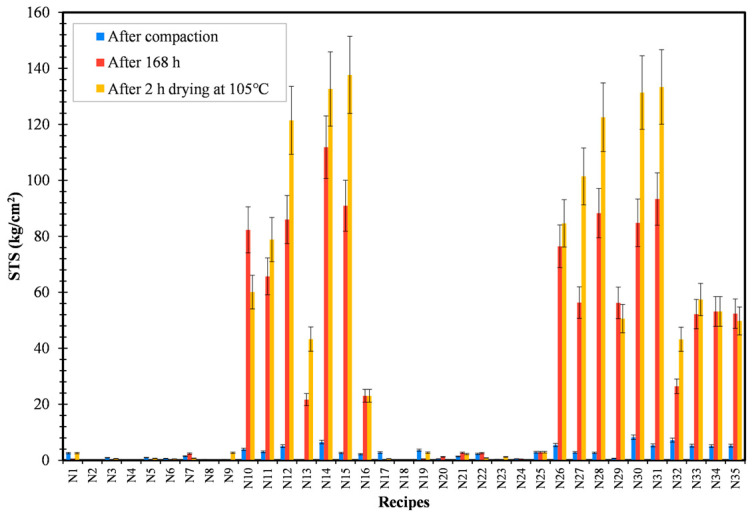
Variation of STS for developed recipes.

The mechanical strength of three briquettes from each recipe was measured using hydraulic compression testing and the results were tabulated. Recipe R5 (sodium silicate), being the best binder material to provide briquettes with the highest dry strength, cannot withstand the cracking and disintegration of briquettes during the reduction process [19]. Hence, it is of utmost importance to add organic binders (Kempel or CB6) with inorganic binders such as sodium silicate. The last three recipes (N33, N34, and N35) were produced under similar conditions so as to check for the reproducibility of the experiment. On comparing recipes N1–N8 with respect to N9–N16, the addition of 5 wt.% water into the recipes gave dominant strength to the recipes. The effect of compaction pressure (50 kN or 200 kN) can be inferred by comparing recipes N1–N16 with respect to N17–N32, respectively. It can be confirmed that the CCS and STS of the recipes were least affected by the change in compaction pressure. Finally, from the strength plot results (Figure 4 and Figure 5), it can be confirmed that the recipes N14/N30 (CB6 + sodium silicate) and N15/N31 (Kempel + sodium silicate) were the best binder combinations and possess the highest CCS and STS out of all recipes. Sweet spot analysis is required to identify the combined effect of compaction pressure, moisture percentage, and wt.% of the binders in each of the recipes. Moreover, the occurrence of the sweet spot will confirm the optimum condition to produce briquettes with the highest compressive and splitting tensile strength.

**Figure 6:**
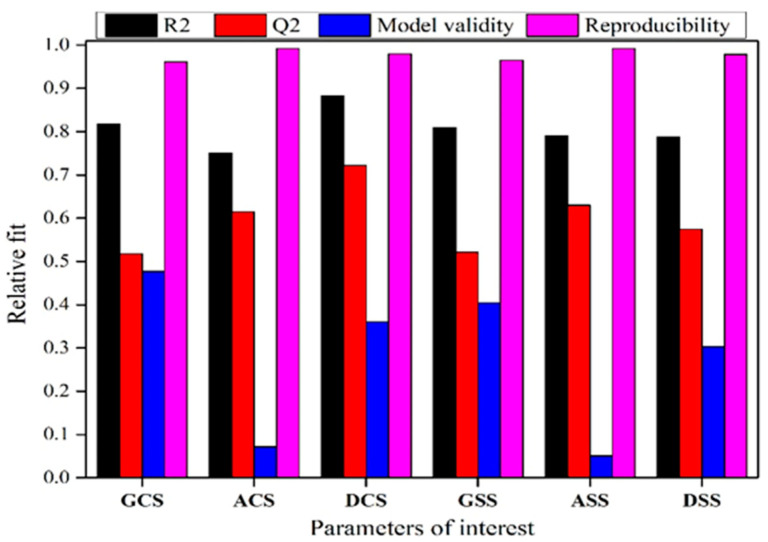
Summary of model fit for all intended responses.

To check for the validity of the model and to check for the credibility of the results obtained from MODDE analysis, a summary of the fit plot has to be evaluated. A summary of the fit plot for all of the intended responses was constructed (Figure 6) and it was observed that each individual response parameter met the pre-requisite condition, which confirms that the results obtained are credible for further analysis. R2 (requisite, R2 > 0.5) indicates whether the model is a good fit to the data, Q2 (requisite, Q2 > 0.5) specifies the extent of prediction power, model validity (requisite, model validity > 0.25) examines whether the model error is smaller than the experimental error, and reproducibility account for small experimental errors [20].

**Figure 7:**
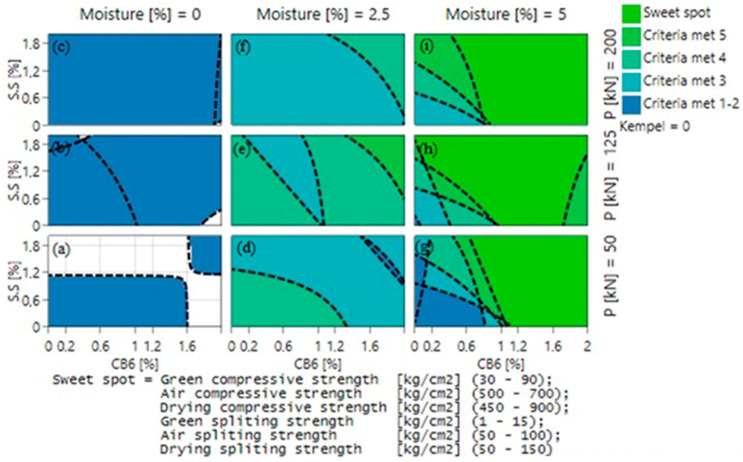
Sweet spot plot when CB6 and sodium silicate (S.S) were used as binder material at: (**a**) 0% moisture, 50 kN pressure (**b**) 0% moisture, 125 kN pressure (**c**) 0% moisture, 200 kN pressure (**d**) 2.5% moisture, 50 kN pressure (**e**) 2.5% moisture, 125 kN pressure (**f**) 2.5% moisture, 200 kN pressure (**g**) 5% moisture, 50 kN pressure (**h**) 5% moisture, 125 kN pressure (**i**) 5% moisture, 200 kN pressure.

**Figure 8:**
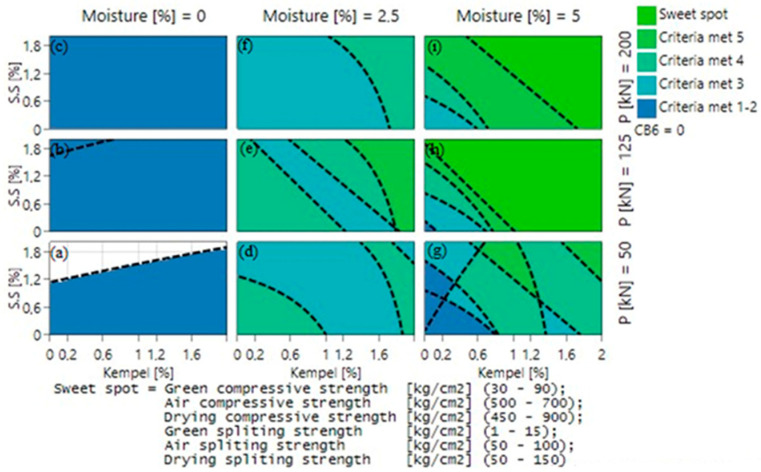
Sweet spot plot when Kempel and sodium silicate (S.S.) were used as binder material at: (**a**) 0% moisture, 50 kN pressure (**b**) 0% moisture, 125 kN pressure (**c**) 0% moisture, 200 kN pressure (**d**) 2.5% moisture, 50 kN pressure (**e**) 2.5% moisture, 125 kN pressure (**f**) 2.5% moisture, 200 kN pressure (**g**) 5% moisture, 50 kN pressure (**h**) 5% moisture, 125 kN pressure (**i**) 5% moisture, 200 kN pressure.

In order to optimize all of the individual responses and obtain the highest compressive and splitting tensile strength, the occurrence of a sweet spot must be analyzed when each parameter is varied. Minimum, maximum, and targeted value for all responses (GCS, GSS, DCS, DSS, ACS, and ASS) was inputted into the MODDE software (version 13) and the consequent sweet spot plot was examined. Figure 7 and Figure 8 illustrate the occurrence of a sweet spot when CB6 + S.S (sodium silicate) and Kempel + S.S are used as a binder, respectively. The six required criteria that need to be satisfied for obtaining a sweet spot are shown in Table 5. These criteria were selected with respect to a previous research work that was carried out with mill scale briquetting [21].

The effect of compaction pressure can be inferred from the vertical blocks of Figure 7a,b,c (likewise Figure 7d,e,f and Figure 7g,h,i) and Figure 8a,b,c (likewise Figure 8d,e,f and Figure 8g,h,i). It can be inferred from Figure 7 that more criteria are met while going up the plot, which confirms that the rise of compaction pressure is preferable for good quality briquettes. Similarly, the effect of moisture content can be deduced from the horizontal blocks of Figure 7a,d,g (likewise Figure 7b,e,h and Figure 7c,f,i) and Figure 8a,d,g (likewise Figure 8b,e,h and Figure 8c,f,i). Results confirmed that while going from left to right, i.e., on increasing moisture content, more criteria are met. With increasing moisture content from 0 to 5%, the number of criteria met was found to increase and finally reaching the sweet spot, where all criteria are met, at a certain binder composition. The most crucial parameter that affects the briquette strength is moisture content, as it is not possible to obtain a sweet spot with 0 or 2.5% of moisture content, but only with 5%, whereas in relation to compaction pressure the sweet spot can be obtained even at lower compaction pressure of 50 kN with 5% moisture content. It can also be ascertained from Figure 7 and Figure 8 that CB6, when in comparison with Kempel, can provide the required briquette strength even at a lower compaction pressure of 50 kN.

Hence, from the sweet spot plots, in both cases, it can be ascertained that the optimized condition for achieving the intended strength is to produce briquettes with 5% moisture content and select the lowest possible binder content according to the compaction pressure. To derive a quite good comparison between the produced briquettes in two cases, a compaction pressure of 125 kN was selected for the briquetting purpose with 1.5 wt.% of organic binder (either CB6 or Kempel) and 0.5 wt.% of inorganic binder in the mixture. Minor additions of inorganic binder in the mix were made so as to achieve dominant hot strength, since most of the organic binders decompose when subjected to high temperature [22,23].

### 3.4. Reduction Results

According to available literature [24,25], reduction temperature for hydrogen reduction should be between 600 °C and 1000 °C. In this work, the reduction temperature set to 950 °C, which is close to the highest applied temperature in a commercial direct reduction process. Fixing the reduction temperature to 950 °C will help briquettes to achieve 100% metallization within a limited amount of time. Therefore, the reduction was performed non-isothermally with H_2_ at a heating rate of 20 °C/min up to 950 °C. Two of the best recipes with the highest strength, according to the sweet spot plots, were selected for the reduction tests and are shown in Table 6.

**Figure 9:**
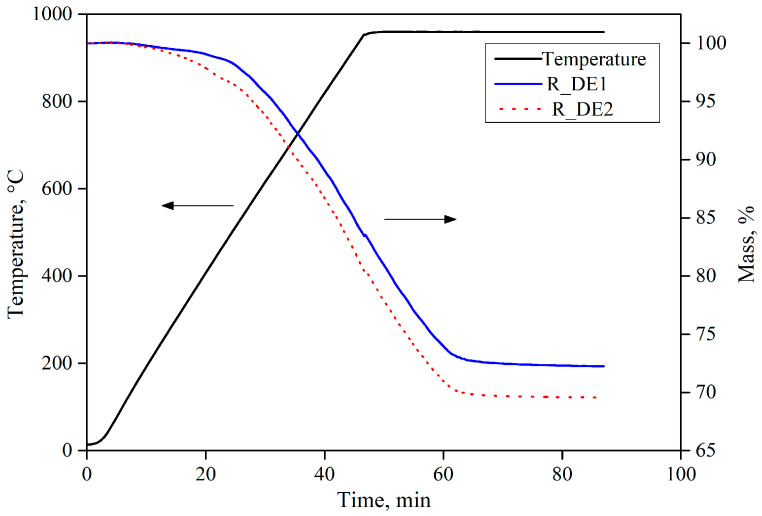
Reduction curves for the developed recipes.

By analyzing the reduction curves of the developed recipes (see Figure 9), it can be ascertained that 100% reduction extent was achieved only after 1 h, with respect to the selected reduction set-up. Furthermore, the mass loss percentage was calculated by weighing the sample before and after the reduction process with hydrogen. Total mass loss % for the developed recipes R_DE1 and R_DE2 was found to be 27% and 30%, respectively.

**Figure 10:**
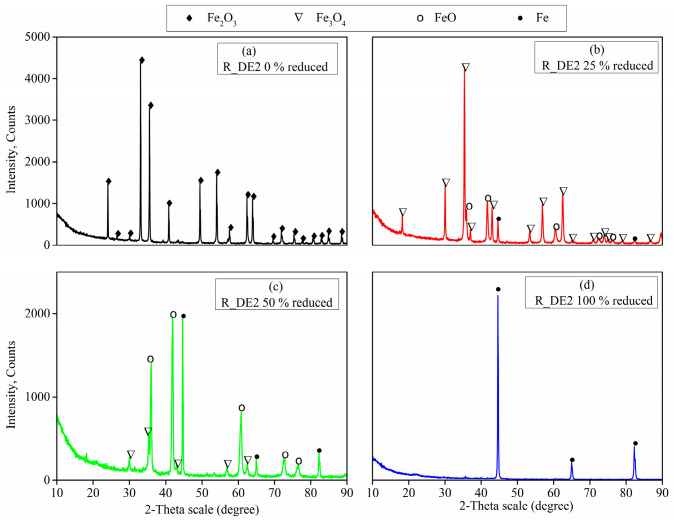
XRD plots for the recipe R_DE2 with the rise in reduction extent.

Since both organic binders (CB6 and Kempel) possessed similar reduction curves with minor deviation in mass loss percentage, it is plausible to do the interrupted test for only one recipe. Both recipes, as shown in Table 6, that were selected for the reduction tests were analyzed using XRD technique (see Figure 10, which shows the development of metallic iron peaks for recipe R_DE2). The process of conversion of iron oxides into metallic iron with the rise in reduction extent is of major interest. Observance of iron oxides in the form of magnetite and hematite was obtained from the peaks of the XRD plots of the raw sample before reduction, as shown in Figure 10a. Increasing the reduction extent to 25% resulted in the formation of wusite and a small amount of iron content could also be confirmed from the XRD plot, as shown in Figure 10b. Further rise in reduction extent gave rise to increased formation of wusite and iron, thereby decreasing the amount of iron oxide, see Figure 10c. In addition, the magnitude of the peaks of iron oxide were also found to decrease with a rise in reduction extent. At 100% reduction extent, as shown in Figure 10d, all of the oxides were reduced to metallic iron and the XRD results for all of the four recipes seemed to be the same with minor variation in phase fractions.

**Figure 11:**
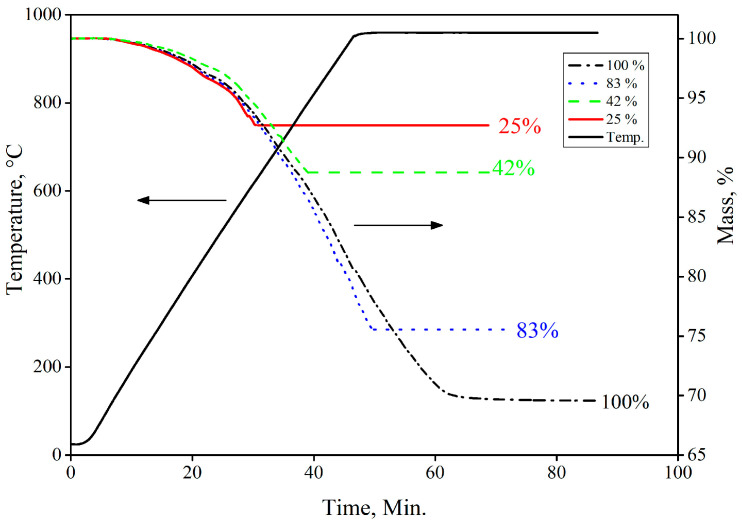
Interrupted reduction test for the recipe with CB6 as binder material.

Interrupted reduction tests on briquettes containing CB6, as the organic binder, were conducted to infer the phase composition and strength variation at 25%, 50%, 90%, and 100% reduction extent. Figure 11 shows the interrupted reduction curves for recipe R_DE2 (with respect to Table 6).

As shown in Table 7, the strength was significantly reduced when the briquettes were heated up to 950 °C and it gradually increased with the reduction extent. This trend can be attributed to the decomposition of organic binders, thereby expelling the volatile matter from the briquette and reducing the strength. By increasing the reduction degree, the metallic iron increased and could be sintered to improve the mechanical strength. Despite of lower strength when in comparison with briquettes before reduction, all of the reduced briquettes were able to possess the required strength (15–20 kg/cm^2^), as suggested by [10]. A gradual rise in mass loss percentage can also be deduced from the interrupted reduction test results, which confirms the increased removal of carbon and other volatile matter from the briquettes with an increase in the reduction extent.

### 3.5. Upscaling Results

By analyzing the mechanical properties and the reduction characteristics of the previously produced briquettes, a combination of 1.5 wt.% of Kempel with 0.5 wt.% of sodium silicate was selected as the binder for the large-scale agglomerate production using an extruder. To obtain a good material fluidity in the extruder, the moisture content of pellet fines mixture with binder was adjusted at 10 wt.%. Therefore, the extruded recipe (R_DE1-extruded) consisted of 88 wt.% pellet fines, 1.5 wt.% Kempel, 0.5 wt.% sodium silicate, and 10 wt.% water, and the extruded briquette had a diameter of 20 mm. By feeding the prepared 8 kg of the recipe at a constant rate, the temperature developed in the pre-compaction and final compaction area was found to be in the range of 30–38 °C.

**Figure 12:**
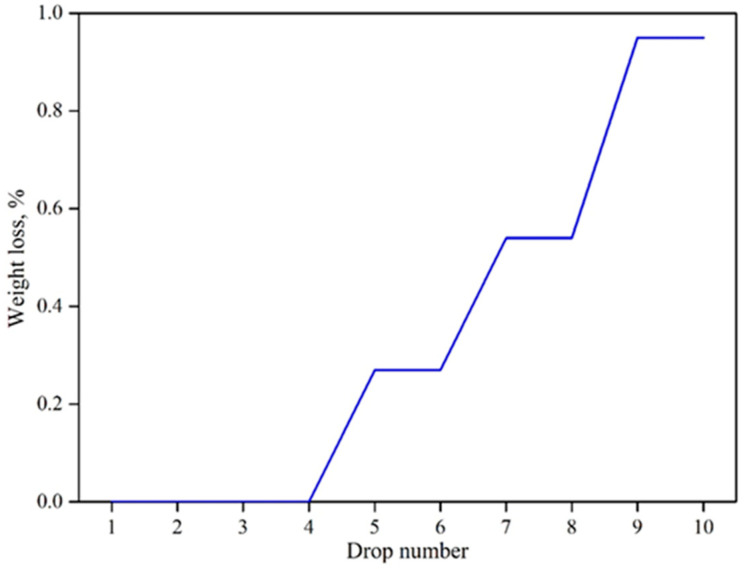
Weight loss percentage versus each drop number.

It is necessary to determine the mechanical strength of the developed briquettes, since mechanical strength is a crucial parameter to consider in order to ensure minimum disintegration caused by the abrasion and impact while loading the briquettes into the shaft furnace. Dropping the developed briquettes from 2 m above the ground into a steel plate was the drop test procedure used to determine the disintegration rate. From Figure 12, it can be ascertained that the recipe R_DE1 can show excellent disintegration resistance with a weight loss percentage of 0.95 wt.%, after sustaining 10 drops. Furthermore, the extruded briquettes were compared with the manual hand-pressed briquettes by scrutinizing the magnitude of cold compressive strength. Table 8 depicts the CCS of the extruded and hand-pressed briquettes before and after reduction. This confirms that the upscaling using an extruder is promising since the briquettes were able to possess the required strength before (60–100 kg/cm^2^) and after reduction (15–20 kg/cm^2^), as suggested by [10]. Furthermore, when comparing the densities of the extruded briquettes and the briquettes produced by the hydraulic press at a compaction pressure of 200 kN, it was found that the extruded briquettes had a lower density (3.16 g/cm^3^) and proved to be more porous than the hydraulic pressed briquettes with a density of 3.85 g/cm^3^.

**Figure 13:**
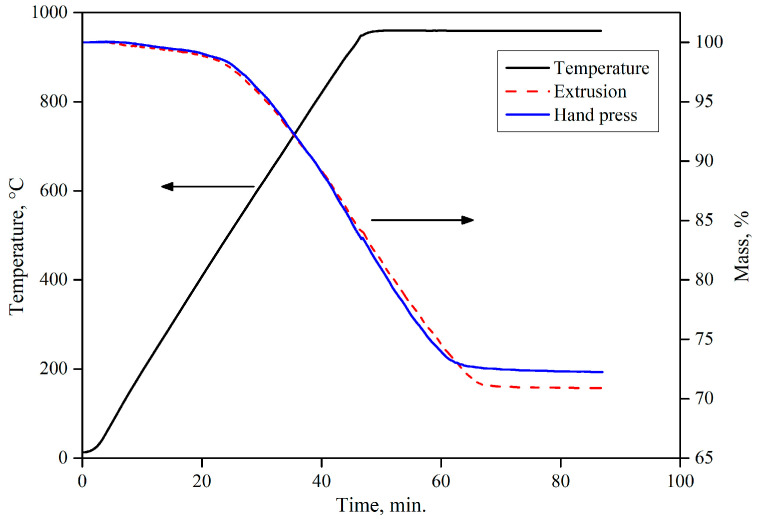
Reduction curves for the extruded and hand-pressed briquette.

Reduction curves of the extruded briquettes, as shown in Figure 13, were compared to the lab-scale produced briquettes. Both briquettes (hydraulic pressed and extruded) depicted a similar trend in reduction behavior. However, the extruded briquettes possessed superior reduction characteristics in terms of reduction rate after the temperature exceeded 500 °C because of the porous structure, which was the result of a lower compaction pressure (in the range of 10–15 kN) during the production. This porous structure made it easy for the reducing gas to pass through the briquette and a high reduction rate was, thus, acquired.

## 4. Conclusions

Efficient utilization of inevitably generated pellet fines in form of briquettes for steel production, along with a need to shift from inorganic binders to organic binders, are the main problems addressed in this study. The current study concentrates on the potential of using novel organic binders for recycling pellet fines during hydrogen-based steel production. The major conclusions inferred from this work are as follows:

- Addition of binders showed a significant improvement in the mechanical strength of the briquettes. Screening of binders identified lignosulfonate, CB6, and Kempel as the best organic binders, in addition to sodium silicate as an in-organic binder.
- Increasing the compaction pressure from 50 kN to 200 kN and the moisture content from 0 wt.% to 5 wt.% improved the mechanical strength of the briquettes. With respect to the drying extent, drying in an oven at 105 °C for 2 h (Drying Compressive Strength/Drying Splitting Strength) was found to be dominant when in comparison with briquettes dried in air for 24 h, 96 h, or 168 h.
- Design of Experiments analysis showed that the best combination to work with is 1.5 wt.% of organic binder (CB6 or Kempel) with 0.5 wt.% of inorganic binder (sodium silicate) with 5 wt.% moisture content in the briquette that is being produced at 125 kN compaction pressure.
- Reducing briquettes in a hydrogen atmosphere with a constant heating rate of 20 °C/min, up to 950 °C, gave promising strength results to the briquettes even after 100% reduction. Interrupted test results affirmed that the reduction extent must be around 90% in order to meet the pre-requisite strength for the briquettes after reduction (15–20 kg/cm^2^). Furthermore, TG analysis showed a gradual rise in mass loss percentage with increasing reduction extent.
- Upscaling using an extruder gave rise to briquettes with the pre-requisite strength before and after reduction. In addition to high strength, extruded briquettes possessed better reduction characteristics due to a highly porous structure. Other promising organic binders such as CB6 and lignosulfonate are recommended to be tested using extruder upscaling in future studies.

With the knowledge of the best combination of organic and inorganic binder to use, this work can be extended to investigate the effect of the addition of biocarbon to the briquettes and its reduction behavior. The addition of biocarbon offers several advantages to steel production, thereby replacing coal as a carbon-bearing material. Furthermore, it helps in slag foaming and reduced electrode consumption during steel production. Hence, as a continuous work, the effect of the addition of biocarbon and its reduction behavior when in contact with hydrogen gas will be explored in upcoming work.

## Figures and Tables

**Table 1:** Selected binders for briquetting.

Selected Binders	Type	Composition	Source
Kempel	Organic	Anionic Polyacrylamide	Kemira, Helsinki, Finland
Lignin	Organic	C_81_H_92_O_28_	Commercial product
Lignosulfonate	Organic	C_20_H_24_Na_2_O_10_S_2_	Borregaard, Sarpsborg, Norway
Starch	Organic	(C_6_H_10_O_5_)_n_	Commercial product
Alcotac CB6	Organic	Polyacrylate (C_17_H_18_O_6_S)	BASF, Heidelberg, Germany
Alcotac FE14	Organic	Anionic Polyacrylamide	BASF, Heidelberg, Germany
Sodium silicate trihydrate	Inorganic	Na_2_SiO_3_·3H_2_O	Commercial product

**Table 2:** Abbreviations and definition.

Parameters	Abbreviation	Definition
Green Compressive Strength	GCS	Compressive strength just after production
Air compressive strength	ACS	Compressive strength after air drying for 7 days
Drying compressive strength	DCS	Compressive strength after over-drying for 2 h at 105 °C
Green Splitting Strength	GSS	Splitting strength just after production
Air splitting strength	ASS	Splitting strength after air drying for 7 days
Drying splitting strength	DSS	Splitting strength after over-drying for 2 h at 105 °C

**Table 3:** Pre-designed recipes for screening of binders.

Material	Recipe No.							
	R0	R1	R2	R3	R4	R5	R6	R7
Pellet fines	100	99	99	99	99	99	99	99
CB6	0	1	0	0	0	0	0	0
FE14	0	0	1	0	0	0	0	0
Kempel	0	0	0	1	0	0	0	0
Starch	0	0	0	0	1	0	0	0
Sodium Silicate	0	0	0	0	0	1	0	0
Lignin	0	0	0	0	0	0	1	0
Lignosulfonate	0	0	0	0	0	0	0	1

**Table 4:** Representative recipes to be produced.

Recipe No.	Pellet Fines, wt.%	CB6, wt.%	Kempel, wt.%	Sodium Silicate, wt.%	Moisture, wt.%	Compaction Pressure, kN
N1	100	0	0	0	0	50
N2	98	2	0	0	0	50
N3	98	0	2	0	0	50
N4	96	2	2	0	0	50
N5	98	0	0	2	0	50
N6	96	2	0	2	0	50
N7	96	0	2	2	0	50
N8	94	2	2	2	0	50
N9	95	0	0	0	5	50
N10	93	2	0	0	5	50
N11	93	0	2	0	5	50
N12	91	2	2	0	5	50
N13	93	0	0	2	5	50
N14	91	2	0	2	5	50
N15	91	0	2	2	5	50
N16	89	2	2	2	5	50
N17	100	0	0	0	0	200
N18	98	2	0	0	0	200
N19	98	0	2	0	0	200
N20	96	2	2	0	0	200
N21	98	0	0	2	0	200
N22	96	2	0	2	0	200
N23	96	0	2	2	0	200
N24	94	2	2	2	0	200
N25	95	0	0	0	5	200
N26	93	2	0	0	5	200
N27	93	0	2	0	5	200
N28	91	2	2	0	5	200
N29	93	0	0	2	5	200
N30	91	2	0	2	5	200
N31	91	0	2	2	5	200
N32	89	2	2	2	5	200
N33	94.5	1	1	1	2.5	125
N34	94.5	1	1	1	2.5	125
N35	94.5	1	1	1	2.5	125

**Table 5:** Criteria that need to be satisfied for obtaining sweet spot.

Criteria	Parameters of Interest	Required Value Range (kg/cm^2^)
1	Green Compressive Strength (GCS)	30–90
2	Air Compressive Strength (ACS)	500–700
3	Drying Compressive Strength (DCS)	450–900
4	Green Splitting Strength (GSS)	1–15
5	Air Splitting Strength (ASS)	50–100
6	Drying Splitting Strength (DSS)	50–150

**Table 6:** Selected recipes for reduction analysis and XRD.

Recipes	Pellet Fines, wt.%	Kempel, wt.%	CB6, wt.%	Sodium Silicate, wt.%	Moisture Content, wt.%
R_DE1	93	1.5	0	0.5	5
R_DE2	93	0	1.5	0.5	5

**Table 7:** Variation of strength and reduction extent for the recipe R_DE2.

Reduction Extent	Mass Loss, %	Strength, kg/cm^2^
0%	0	122
25%	7	8
50%	10.5	16.5
90%	26	31
100%	30	33

**Table 8:** Mechanical strength of the extruded and hydraulic press briquette R_DE2.

Parameters	Extruded Briquettes	Hydraulic Pressed Briquettes
Strength before reduction, kg/cm^2^	103	122
Strength after reduction, kg/cm^2^	70	81
Mass loss, %	33	31
